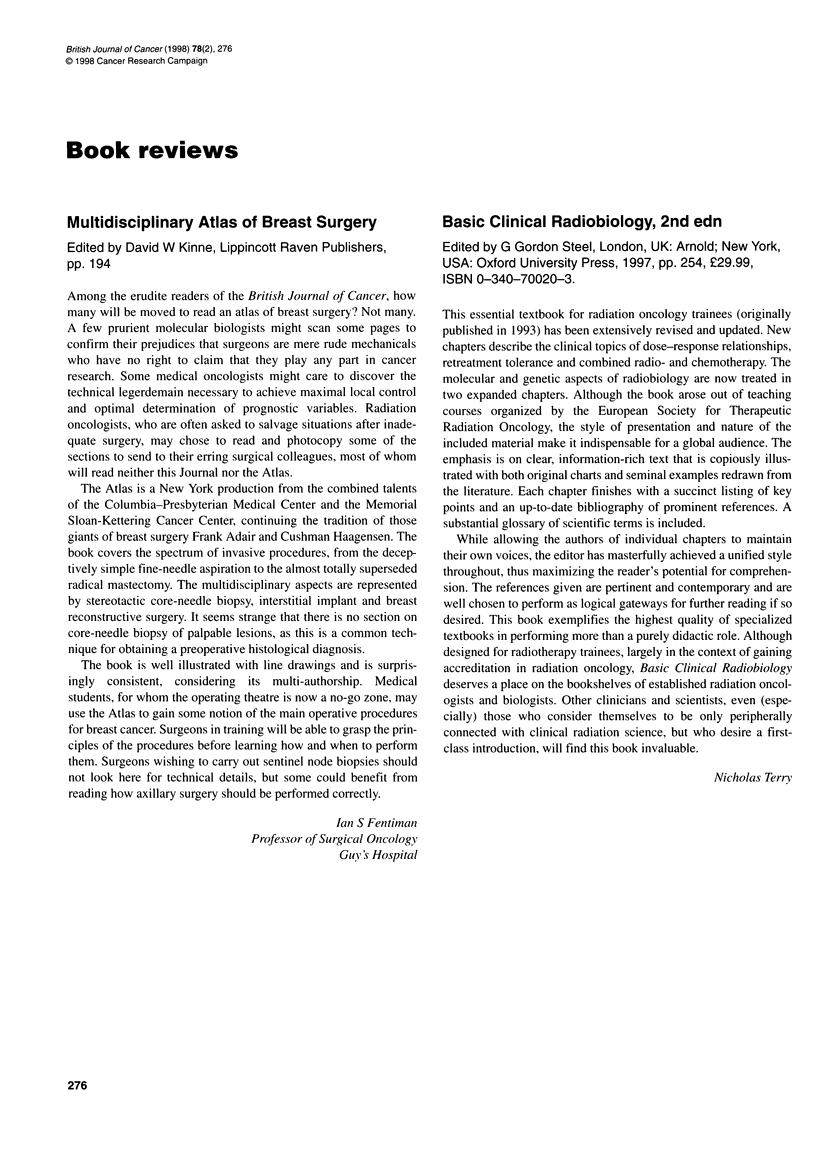# Multidisciplinary Atlas of Breast Surgery

**Published:** 1998-07

**Authors:** Ian S Fentiman


*British Journal of Cancer* (1998) **78**(2), 276
© 1998 Cancer Research Campaign

### Book reviews

### Multidisciplinary Atlas of Breast Surgery

Edited by David W Kinne, Lippincott Raven Publishers, pp. 194

Among the erudite readers of the *British Journal of Cancer*, how many will be moved to read an atlas of breast surgery? Not many. A few prurient molecular biologists might scan some pages to confirm their prejudices that surgeons are mere rude mechanicals who have no right to claim that they play any part in cancer research. Some medical oncologists might care to discover the technical legerdemain necessary to achieve maximal local control and optimal determination of prognostic variables. Radiation oncologists, who are often asked to salvage situations after inadequate surgery, may chose to read and photocopy some of the sections to send to their erring surgical colleagues, most of whom will read neither this Journal nor the Atlas.

The Atlas is a New York production from the combined talents of the Columbia–Presbyterian Medical Center and the Memorial Sloan-Kettering Cancer Center, continuing the tradition of those giants of breast surgery Frank Adair and Cushman Haagensen. The book covers the spectrum of invasive procedures, from the deceptively simple fine-needle aspiration to the almost totally superseded radical mastectomy. The multidisciplinary aspects are represented by stereotactic core-needle biopsy, interstitial implant and breast reconstructive surgery. It seems strange that there is no section on core-needle biopsy of palpable lesions, as this is a common technique for obtaining a preoperative histological diagnosis.

The book is well illustrated with line drawings and is surprisingly consistent, considering its multi-authorship. Medical students, for whom the operating theatre is now a no-go zone, may use the Atlas to gain some notion of the main operative procedures for breast cancer. Surgeons in training will be able to grasp the principles of the procedures before learning how and when to perform them. Surgeons wishing to carry out sentinel node biopsies should not look here for technical details, but some could benefit from reading how axillary surgery should be performed correctly.

*Ian S Fentiman*
*Professor of Surgical Oncology*
*Guy's Hospital*